# Cutaneous Localization of Classic Hodgkin Lymphoma Associated with Mycosis Fungoides: Report of a Rare Event and Review of the Literature

**DOI:** 10.3390/life11101069

**Published:** 2021-10-11

**Authors:** Magda Zanelli, Stefano Ricci, Francesca Sanguedolce, Andrea Palicelli, Enrico Farnetti, Alessandro Tafuni, Maurizio Zizzo, Riccardo Valli, Maria Isabel Alvarez De Celis, Alberto Cavazza, Caterina Longo, Stefano Ascani

**Affiliations:** 1 Pathology Unit, Azienda USL-IRCCS di Reggio Emilia, 42123 Reggio Emilia, Italy; stefano.ricci@ausl.re.it (S.R.); andrea.palicelli@ausl.re.it (A.P.); Alessandro.tafuni@unipr.it (A.T.); riccardo.valli@ausl.re.it (R.V.); alberto.cavazza@ausl.re.it (A.C.); 2 Pathology Unit, Policlinico Riuniti, University of Foggia, 71122 Foggia, Italy; francesca.sanguedolce@unifg.it; 3 Molecular Biology Laboratory, Azienda USL-IRCCS di Reggio Emilia, 42123 Reggio Emilia, Italy; enrico.farnetti@ausl.re.it; 4 Pathology Unit, Department of Medicine and Surgery, University of Parma, 43121 Parma, Italy; 5 Surgical Oncology Unit, Azienda USL-IRCCS di Reggio Emilia, 42123 Reggio Emilia, Italy; maurizio.zizzo@ausl.re.it; 6 Hematology Unit, Azienda USL-IRCCS di Reggio Emilia, 42123 Reggio Emilia, Italy; alvarezdecelis.mariaisabel@ausl.re.it; 7 Centro Oncologico ad Alta Tecnologia Diagnostica, Azienda USL-IRCCS di Reggio Emilia, 42123 Reggio Emilia, Italy; caterina.longo@ausl.re.it; 8 Pathology Unit, Azienda Ospedaliera Santa Maria di Terni, University of Perugia, 05100 Terni, Italy; s.ascani@aospterni.it

**Keywords:** mycosis fungoides, classic Hodgkin lymphoma, skin

## Abstract

Mycosis fungoides and nodal classic Hodgkin lymphoma (cHL) have been reported to occur concurrently or sequentially in the same patient. A long-lasting mycosis fungoides more often precedes the onset of nodal cHL, although few cases of nodal cHL followed by mycosis fungoides have been observed. Skin involvement is a rare manifestation of cHL that may be observed in the setting of advanced disease. The decrease in skin involvement in cHL is mainly due to the improved therapeutic strategies. The concurrent presence of mycosis fungoides and cutaneous localization of classic Hodgkin lymphoma represents a very uncommon event, with only two cases reported so far. Herein, we describe the case of a 71-year-old man, with a history of recurrent nodal cHL, who developed MF and, subsequently, the cutaneous localization of cHL. The clinicopathological features of the two diseases are described focusing on the main differential diagnoses to be taken into consideration, and a review of the literature is performed.

## 1. Introduction

Classic Hodgkin lymphoma (cHL) is a monoclonal lymphoid neoplasm of B-cell origin, made up of a variable number of neoplastic elements—that is, mononuclear Hodgkin cells and multinucleated Reed–Sternberg cells (RSCs)—in a reactive background with different inflammatory cells [1]. On the basis of both the morphological features of the neoplastic elements and the characteristics of the reactive infiltrate, the disease is divided into four subtypes: nodular sclerosis cHL (NSCHL), lymphocyte-rich cHL (LRCHL), mixed cellularity cHL (MCCHL), and lymphocyte-depleted cHL (LDCHL) [1,2]. Although the clinical manifestations vary among the different subtypes, cHL is mainly a nodal disease, with often mediastinal involvement [3]. Extra-nodal sites such as spleen, liver, lungs, or bones may be affected secondarily in the disease course.

Primary extra-nodal cHL represents an extremely uncommon event [4,5].

In cHL, cutaneous involvement is rare (<1%), represents an advanced disease stage, and is the result of direct, lymphatic, or hematogenous spread [6,7,8,9]. Rare cases of cHL arising primarily in the skin without concomitant nodal disease have been reported [10]. Due to its rarity, extreme caution is advised before making a diagnosis of primary cutaneous cHL. Other entities more commonly presenting in the skin and histologically mimicking cHL need to be ruled out. In particular, lymphomatoid papulosis (LyP), cutaneous anaplastic large cell lymphoma (C-ALCL), and Epstein–Barr virus positive mucocutaneous ulcer (EBVMCU) need to be taken into consideration before diagnosing primary cutaneous cHL [11,12,13,14].

Mycosis fungoides (MF) is the most common type of skin lymphoma, accounting for almost 50% of all primary cutaneous lymphomas. In the classic form, MF is an epidermotropic lymphoma made up of small to medium-sized T lymphocytes with cerebriform nuclei, often with a T-helper phenotype (CD4-positive), although T-cytotoxic variants are not infrequent [1]. The classical clinical presentation is with erythematous patches, commonly in sun-protected areas, slowly progressing to plaques and, eventually, tumors over years or decades [1,14].

Patients with MF have an increased risk of developing a second nonhematologic or hematologic malignancy including cHL [15,16,17,18,19]. cHL patients as well are known to be at higher risk for second malignancy, including non-Hodgkin lymphomas [20,21].

More frequently, MF precedes the occurrence of cHL [15,17,18,19], although even a different sequence of events is reported [22,23,24]. 

Herein, we present a very unusual case of skin involvement by two types of lymphomas: cHL and MF. After a history of recurrent nodal cHL, the patient developed MF and, subsequently, cutaneous localizations of cHL. Presenting this case, we aim to focus in particular on the main clinicopathological differential diagnoses, which need to be evaluated.

Based on the PRISMA guidelines, we carried out a systematic search on Pubmed/MEDLINE, Web of Science, Scopus, EMBASE, and Cochrane Library using the search terms “Mycosis fungoides” and “Classic Hodgkin lymphoma”. Our literature search identified only two cases in which MF and cHL with skin involvement were co-existent [25,26].

## 2. Case Presentation

A 71-year-old man was first diagnosed in 2010 with cHL, which manifested as multiple abdominal lymphadenopathy (stage IIA) and was treated with an ABVD (adriamycin, bleomycin, vinblastine, dacarbazine) chemotherapy scheme.

The disease relapsed a total of four times: in 2012 with a single axillary lymphadenopathy (stage IA) treated with radiotherapy (40 Gy/fraction for 20 times); in 2014 as stage IIA disease treated with an IGEV (ifosfamide, gentamicin, vinorelbine) scheme, followed by autologous bone marrow transplant (ABMT) with FEAM (fotemustine, etoposide, cytarabine, melphalan) conditioning regimen; and in 2018 and 2019 with cutaneous localizations treated with the anti CD30 monoclonal antibody (brentuximab vedotin) and anti-PD-L1 (nivolumab), respectively.

Despite complete regression of the skin lesions, at last follow-up (July 2021), multiple lymphadenopathies were identified, and the patient is still on nivolumab.

MF first appeared in 2014, shortly after the second cHL relapse, and it was treated with PUVA (psoralen and ultraviolet A) therapy with complete remission. MF relapsed in 2020, and PUVA therapy resulted in partial improvement.

**Figure 1:**
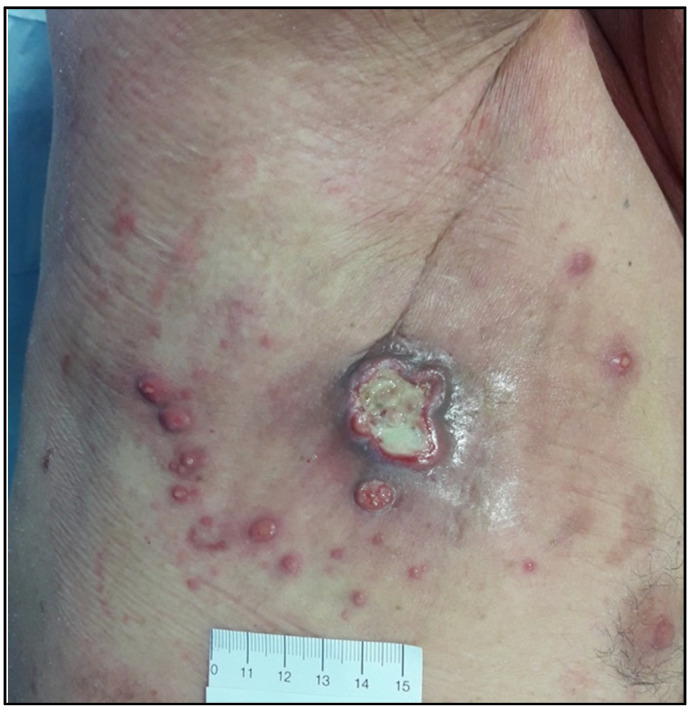
Cutaneous manifestation of cHL: a large, ulcerated nodule plus multiple smaller papules in the axillary region (original image from Dr. M.I.A.d.C).

The cutaneous manifestations of cHL consisted of an ulcerated nodule and itching papules in the axillary region, very close to MF involved skin areas (Figure 1).

Multiple papules on the limbs, neck, umbilical region, and back were also present. The papules were partially ulcerated and showed a crowding pattern; between discrete lesions, erythematous scaly patches were also noted.

**Figure 2:**
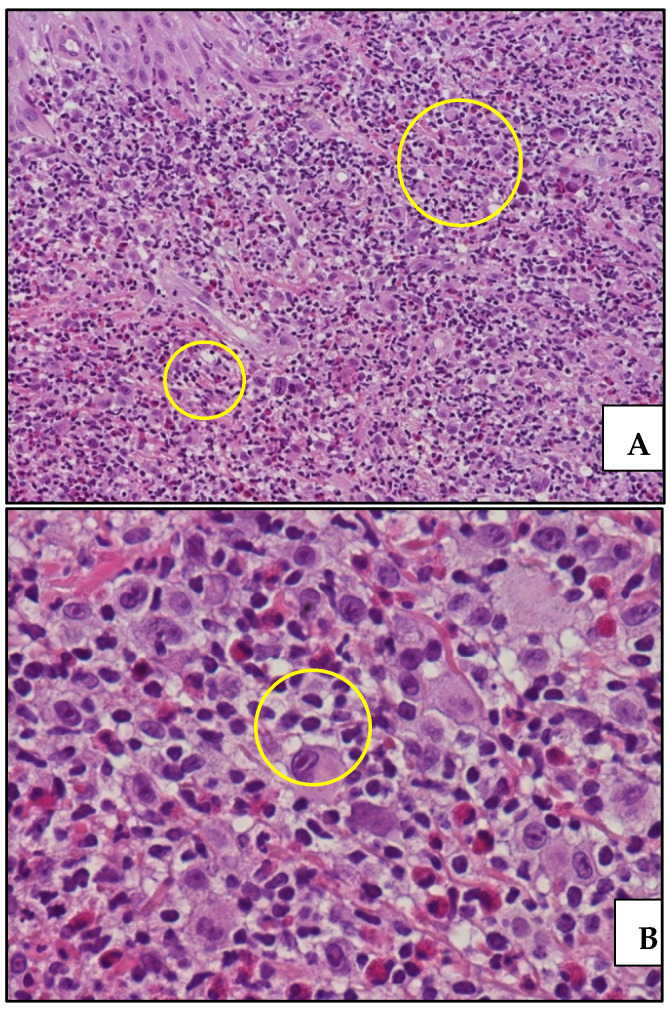
(**A**) Histology of cutaneous localization of cHL: medium power view showing a polymorphic dermal infiltrate containing sparse, atypical, large-sized cells (yellow circles) (hematoxylin and eosin staining, 200× magnification; original image from Dr. M. Zanelli; (**B**) high-power view highlighting dispersed atypical cells with prominent nucleolus (yellow circle) within an inflammatory background rich in eosinophils (hematoxylin and eosin staining, 400× magnification; original image from Dr. M. Zanelli).

**Figure 3:**
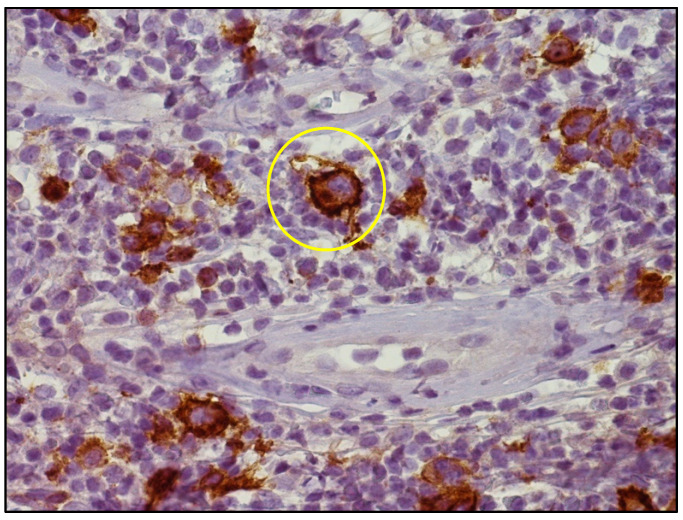
High-power view of CD30 immunostaining highlighting atypical cells with membranous and dot-like paranuclear positivity (yellow circle) (CD30 immunostaining, Ventana immunostainer, 400× magnification; original image from Dr. M. Zanelli).

Punch biopsy from a single papule in the axillary region showed a dense dermal infiltrate rich in eosinophils containing dispersed large cells with Hodgkin and RSC morphology expressing CD30 and MUM1/IRF4, weakly positive for PAX5 and negative for CD15 and LCA. In situ hybridization for EBV-encoded RNA (EBER) was negative. Clonality analyses failed owing to poor DNA quality. The histology was consistent with the cutaneous localization of cHL (Figure 2 and Figure 3).

**Figure 4:**
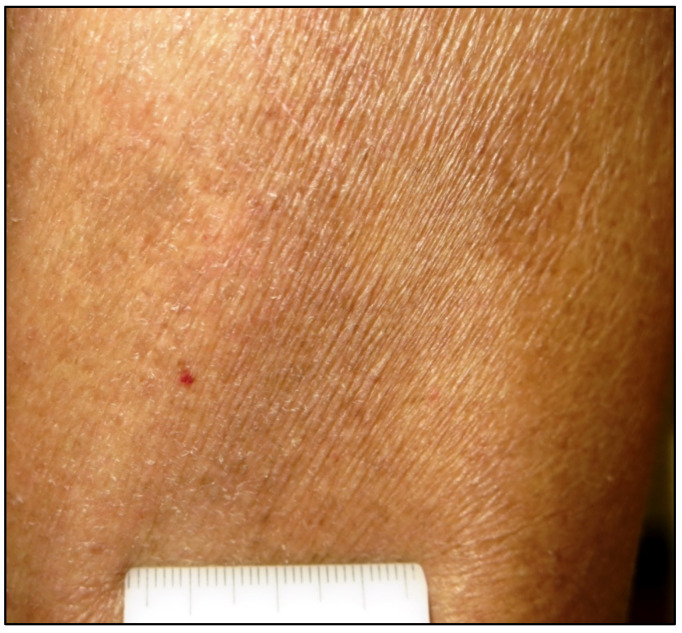
The clinical features of early MF may be rather subtle: a slightly erythematous and finely scaling patch with a wrinkled appearance (original image from Prof. C.L).

MF first appeared in 2014 with rather subtle, slightly erythematous and finely scaling patches on the right arm (Figure 4) and groins.

**Figure 5:**
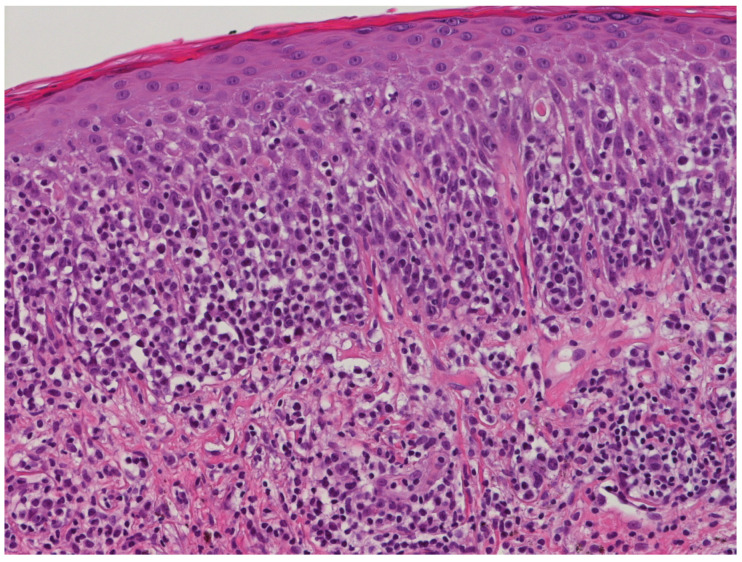
Medium-power view of MF histology: the dermal infiltrate consists of small and medium-sized lymphocytes with evident epidermotropism (hematoxylin and eosin staining, 200×; original image from Dr. M. Zanelli).

**Figure 6:**
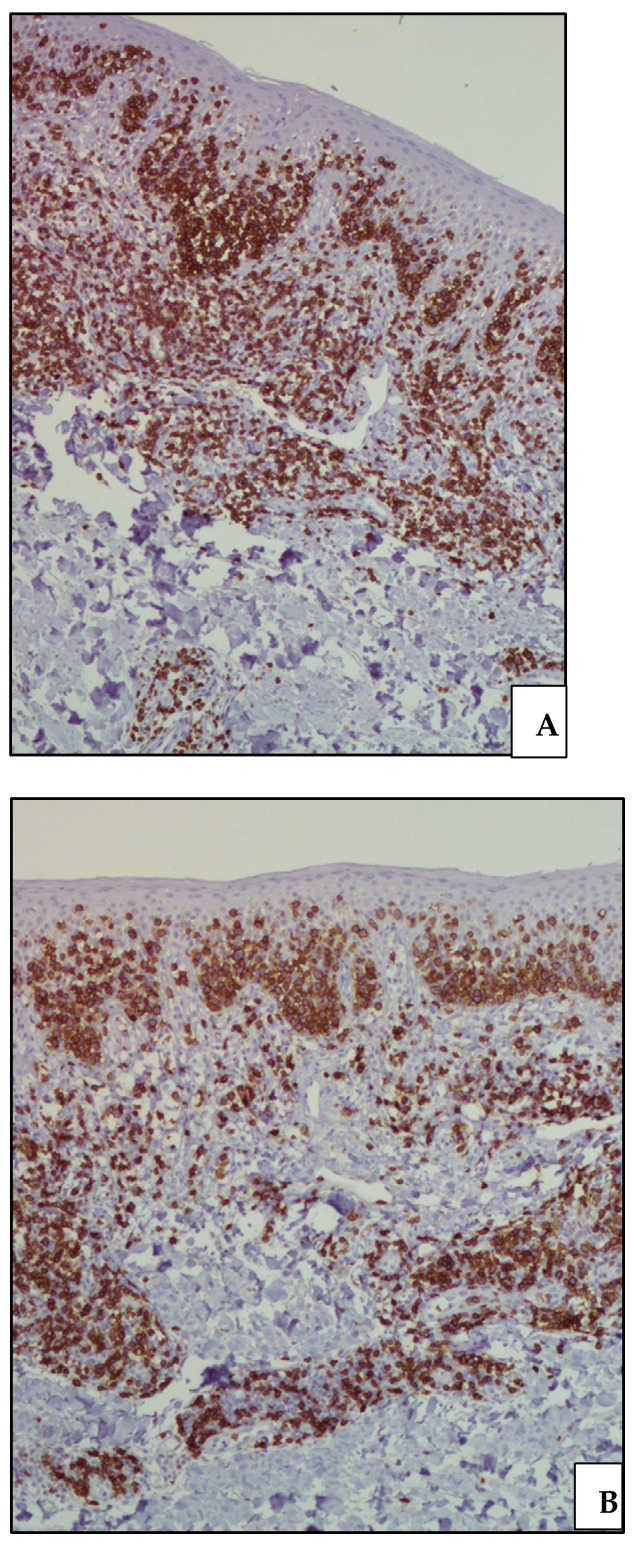
(**A**) Medium-power view of CD3 immunostaining confirming the T-cell nature of the dermal and epidermal lymphoid infiltrate (CD3 immunostaining, Ventana immunostainer, 200× magnification; original image from Dr. M. Zanelli); (**B**) CD8 positivity highlighting the cytotoxic phenotype of the dermal infiltrate with striking epidermotropism (CD8 immunostaining, Ventana immunostainer, 200× magnification; original image from Dr. M. Zanelli).

In 2020, MF relapsed as multiple patches and variably pigmented, infiltrated plaques on trunk and limbs. A punch biopsy of a plaque showed a band-like, lichenoid lymphocytic infiltrate in the superficial dermis with striking epidermotropism (Figure 5 and Figure 6).

The lymphoid cells were small to medium-sized and resulted positive for CD3, CD7, and CD8 and negative for CD5, CD2, CD4, CD30, CD56, CD20, and PAX5. The proliferative fraction (Ki67) was low. T-cell receptor (TCR) gene analysis failed due to poor DNA quality.

The findings were consistent with MF, without any sign of transformation into aggressive T cell lymphoma or concurrent cHL.

## 3. Literature Review: Methods and Results

We performed our literature review adhering to the Preferred Reported Items for Systematic Reviews and Meta-analyses (PRISMA) guidelines. Pubmed/MEDLINE, EMBASE, Scopus, Cochrane Library, and Web of Science (Science and Social Science Citation Index) databases were used in order to search all related literature, using the following non-MeSH/MeSH terms (“mycosis fungoides” and “classic Hodgkin lymphoma”). The search was performed from the inception of the databases until August 2021. The criteria for inclusion were the following: (1) a diagnosis of MF and cHL based on reliable morphological and immunophenotypical features according to the current criteria of World Health Organization (WHO) Classification of Tumours of Haematopoietic and Lymphoid Tissues [1]; (2) co-occurrence of MF and cHL; (3) presence of cutaneous localization of cHL; (4) retrospective, observational case-control studies, case reports and/or series, literature review. The exclusion criteria were as follows: (1) studies not published in English; (2) lack of adherence to the diagnostic criteria for MF and cHL according to current WHO classification [1]. Three independent reviewers (M.Zanelli, FS, and SR) selected and identified the papers on the basis of title, abstract, key words, and full text. From the selected papers, the following information was collected: patient’s age and sex; timing of occurrence of MF and cHL; timing of occurrence of cutaneous involvement by cHL; morphological, immunohistochemical, and molecular features of MF and cHL; therapy and clinical outcome. All collected results were revised by a fourth independent reviewer (S.A). Finally, the search identified an overall number of two articles [25,26].

In the two studies, a total of two cases with co-occurrence of MF and cHL with cutaneous involvement were reported [25,26].

Both patients were males and aged 34 and 63 years, respectively. 

In both cases, a long-lasting MF preceded the occurrence of cHL. In the report by Geldenhuys et al., nodal cHL and cutaneous involvement by cHL appeared simultaneously after a 10-year history of MF [25]. In the report by Chen et al., skin lesions consistent with MF had been present for 3 years before the simultaneous occurrence of cHL in both the lymph node and skin [26]; in particular, in this case, the skin biopsy showed the co-existence of cHL and MF.

In both reports, the cutaneous involvement by cHL developed in skin areas affected by MF.

The histology and immunophenotyping were consistent with the diagnostic criteria for MF and cHL. In the report by Chen et al., molecular analyses were also performed, supporting the diagnosis of both MF and cHL. The T-cell monoclonal nature of MF lesions was confirmed, whereas B-cell clonality was identified in cells microdissected from skin lesions, confirming the diagnosis of cHL [26].

In the two cases, different treatments were administered. In the case by Geldenhuys et al., cHL was treated with a MOPP (mechlorethamine, vincristine, procarbazine, prednisone) scheme with complete remission at last follow-up (11 years later); MF was treated with topical nitrogen mustard and, subsequently, with PUVA therapy with complete regression of cutaneous lesions [25]. In the report by Chen et al., cHL was treated with an ABVD scheme with complete remission; MF was treated with interferon-alpha in combination with low-dose oral methotrexate and the histone deacetylase inhibitor chidamide, achieving complete remission within 6 months.

## 4. Discussion

MF can be observed in association with other hematological and nonhematological diseases [14,15,17,19]. These disorders more often follow MF diagnosis, presenting after a prolonged MF course. However, their onset may even precede or be concomitant with MF. Patients with MF show an increased incidence of a second malignancy, including cHL [15,16,18].

On the other hand, patients with a previous diagnosis of cHL are known to be at increased risk of developing a second tumor, including non-Hodgkin lymphomas [20,21]. There are more frequent reports of cHL following a previous diagnosis of MF than the other way around [15,17,19,22,23,24,25].

A recent meta-analysis of articles published between 1950 and 2019 was performed by Goyal et al. to evaluate the risk of second tumors in patients with cutaneous T-cell lymphomas including predominantly MF. Of the 11,214 patients included in the study, 8.5% developed second malignancies, and of patients with second cancers, 17.8% developed cHL [18].

While examples of concurrent nodal cHL and MF have been frequently reported, the concomitant occurrence of MF and cutaneous localization of cHL represents an extremely rare event. From a literature review, the co-occurrence of MF and cutaneous involvement by cHL has been reported only twice, and in both cases a long-lasting MF preceded the occurrence of cHL [25,26].

In contrast with the two previous reports, in our case, nodal cHL occurred a few years before MF. Although the occurrence of the two diseases in the same patient has been reported several times, only a few reports documented MF occurrence following a previous diagnosis of cHL [22,23,24,25]. 

Unlike the two previous reports in which nodal cHL and cutaneous involvement by cHL developed simultaneously, in our case, MF preceded the cutaneous localization of cHL by a few years.

The cutaneous involvement in cHL is rare, owing to the improvement in treatment strategies. It is generally secondary, occurring in advanced stage nodal disease, as was in our patient [8,14]. Cutaneous manifestations include plaques, papules, and nodules; lesions are often ulcerated. In cHL, cutaneous involvement is usually limited to the drainage region of affected lymph nodes, although generalized lesions may occur [14].

As mentioned above, primary cutaneous cHL represents an extremely uncommon event, and such a diagnosis should be considered only after having ruled out other entities closely simulating cHL. LyP is a recurrent skin-limited disease, presenting with ulcerated papules or nodules at various stages of development. The histology of LyP is extremely variable with multiple histological subtypes; in particular, type A LyP, consisting of large atypical CD30-positive cells with RSC morphology within an inflammatory background, may mimic cHL [1,14].

C-ALCL, a skin-limited lymphoma of T-cell origin, is another mimicker of cHL. Therefore, as pointed out by Cerroni, it is possible that at least some of the cases reported in the past as primary cutaneous cHL may be either LyP or C-ALCL [14].

Another entity to be ruled out before considering primary cutaneous cHL is EBVMCU, usually affecting the oropharynx, skin, and gastrointestinal tract [1,11,12,13,14]. EBVMCU presents as a superficial and circumscribed, often solitary, ulcer, without forming a mass [11,12,13], usually developing in the setting of patients with either age-related or iatrogenic immunosuppression. The diagnosis requires the strict integration of clinical and pathological features, as the histology alone does not allow to exclude cHL.

In our case, the cutaneous localization of cHL occurred in a patient with a history of relapsed nodal cHL; therefore, primary cutaneous cHL was out of question. However, four years before the cutaneous manifestations of cHL, the patient had developed MF; therefore, the differential diagnosis between MF and a cutaneous localization of cHL was essential for therapeutic purposes.

The differential diagnosis may be complicated as, similar to cHL, CD30 expression may be found in neoplastic cells of MF, in particular in the stages of plaques and tumors. CD15 expression by neoplastic elements supports cHL diagnosis; PAX5 positivity in cHL confirms the B-cell origin, whereas the neoplastic cells of MF are of T-cell lineage and, more often, with a T-helper phenotype [1]. In a subset of MF, the phenotype is cytotoxic with CD8 expression. In CD8-positive MF, as was our case, distinction from other more aggressive lymphomas such as, for instance, primary cutaneous aggressive epidermotropic CD8-positive cytotoxic T-cell lymphoma is critical and based mainly on clinical presentation and outcome. Unlike MF, patients with primary cutaneous aggressive epidermotropic CD8-positive cytotoxic T-cell lymphoma present with ulcerated plaques and tumors at disease onset, and prognosis is poor [1,14].

Few cases of cHL may aberrantly express T-cell antigens by immunohistochemistry, making the differential diagnosis with MF even more complicated. In some cases, gene rearrangement studies may represent a further aid, demonstrating T-cell clonality of MF, whereas cHL neoplastic cells are usually negative for the rearrangement of T-cell receptor (TCR) genes [14,27]. Clonal rearrangement of immunoglobulin (IG) genes may be found in cHL, confirming the B-cell origin. Unfortunately, molecular analysis of the entire biopsy specimen may fail to reveal clonality in cutaneous manifestations of cHL owing to the scarce number of neoplastic elements within the inflammatory background. Molecular analysis on microdissected Hodgkin cells and RSCs may reveal B-cell clonality [14]. In the case by Chen et al., molecular analyses were helpful in supporting the diagnosis by revealing the T-cell clonality of MF and B-cell clonality of cHL cells microdissected from skin lesions [26].

The main differential diagnoses are summarized in Supplementary Table S1.

Secondary cutaneous involvement by systemic lymphomas is an event always to be kept in mind, as it may represent the first manifestation of the underlying systemic lymphoma [14,28,29].

The co-occurrence of MF and cHL raises some questions: firstly about the differential diagnosis between relapse of the previously known lymphoma and occurrence of a new one, secondly about the prognostic significance of a secondary lymphoma and, lastly, about a possible common clonal origin of the diseases [26]. In occasional cases, the same clone has been detected in MF and associated lymphomas, while in others, the clone was different [30,31]. 

A role in the occurrence of second hematological malignancies is likely to be played either by the direct toxicity of previous chemotherapy and radiotherapy or by altered immunity associated with the previous lymphoma [22]. An interesting feature observed in both the previous reports as well as in our case is the presence of cHL cutaneous lesions close to skin areas involved by MF.

It might be hypothesized that local immunosuppression by the malignant T-cells of MF may create a favorable immunological environment for the localization of cHL in the skin.

## 5. Conclusions

The present case highlights some interesting points to take into consideration: (a) the occurrence of a secondary lymphoma is not out of the question in patients with a previous history of a different hematological neoplasm; therefore, the differential diagnosis is critical in cases with equivocal morphological or immunophenotypical features; (b) previous treatments (chemotherapy or radiotherapy) may play a role in the development of second lymphomas; (c) patients with relapsing MF have to be carefully evaluated, as not all skin lesions might be manifestations of MF.

## Data Availability

The data presented in this study are available on request from the corresponding author.

## Supplementary Materials

The following are available online at https://www.mdpi.com/article/10.3390/life11101069/s1, Table S1: Differential diagnoses.
