# Supplementary material for: Cutaneous Localization of Classic Hodgkin Lymphoma Associated with Mycosis Fungoides: Report of a Rare Event and Review of the Literature
Source: Life (Basel). 2021 Oct 11;11(10):1069. doi: 10.3390/life11101069 (PMC8537882; doi:10.3390/life11101069)
Supplement: Supplementary file 1 [file life-11-01069-s001.zip › life-1404653-supplementary.pdf]

Table S1. Differential diagnoses

	Clinical Presentation	Extra-cutaneous Sites	Histology	IHC	Genetic Profile	Outcome
**MF**	Patches, plaques, tumors	Lymph nodes, liver, spleen, lungs (in advanced stages)	Lichenoid dermal infiltrate with epidermotropism; small to medium-sized lymphocytes	CD3+ CD2+ CD5+ CD7- CD4+ (more often) CD8- (more often) TCR beta+ TCR gamma- (more often) CLA+ CD30+/-	TCR+	Slow progression over many years; outcome depends on clinical stage
**Cutaneous cHL (usually secondary)**	Papules, plaques, tumors	Lymph nodes and other extra-cutaneous sites involved. Cutaneous cHL occurs in advanced disease	Large-sized cells with Hodgkin and RSC morphology within inflammatory background	CD30+ CD15+/- MUM1+ PAX5+ weak CD20- or variably + CD79alpha- or variably+ LCA- T-cell antigens may be aberrantly + EBER+/-	IGH+ TCR-	Poor
**C-ALCL**	Usually skin-limited disease; often solitary ulcerated tumor	Rare (10% of cases in regional lymph nodes)	Cohesive sheets of cells with anaplastic, pleomorphic or immunoblastic features; sometimes inflammatory background (pyogenic variant); epidermotropism often in DUSP22-IRF4+	CD4+; CD4-CD8+; CD4+CD8+: or null; perforin+ TIA1+ Granz. B+ CD30+ CD15+ (40%) MUM1+ EMA- ALK- PAX5- EBER-	TCR+<br>DUSP22-IRF4+ (25%)	Favorable
**LyP**	Skin-limited disease; multiple necrotic papules and nodules	absent	Different histological patterns:<br>Type A: sparse atypical large CD30+ cells within inflammatory elements;<br>Type B: epidermotropic	CD4+ cells in types A and C;<br>CD8+ cells in types D and E;<br>CD8+ or CD4-CD8-<br>in DUSP22-IRF4	TCR+ in most cases;<br>rare cases DUSP22-IRF4 rearranged	Excellent; usually a recurrent, self-healing disease; increased risk of developing lymphomas

			infiltrate of CD30+ or CD30- small cells simulating MF;<br>Type C: Sheets of large CD30+ cells with scarse inflammation simulating C-ALCL;<br>Type D: epidermotropic infiltrate of small to medium CD8+ CD30+ cells;<br>Type E: angiocentric pattern of small or medium CD8+ CD30+ cells	translocated cases		
**EBVMCU**	Circumscribed, often solitary ulcer in the setting of IS (age-related or iatrogenic)	No (EBVMCU may develop even in oropharynx and GIT)	Polymorphic pattern with sparse atypical large cells with RSC features (simulating cHL); or monomorphic pattern with sheets of cells simulating DLBCL	CD30+<br>PAX5+<br>CD20 variably+<br>CD79a+<br>OCT2+<br>BOB1 variably+<br>MUM1+<br>CD15+(50%)<br>CD10-<br>BCL6-<br>EBER+	IG clonality (50%)<br><br>TCR oligoglonal	Favorable with regression upon reduction or removal of IS cause
**Primary cutaneous CD8+ aggressive epid. cytotoxic T-cell lymphoma**	Generalized skin lesions (ulcerated plaques or tumors)	Visceral sites (lungs, testes, CNS, oral mucosa), lymph nodes rarely affected	Marked pagetoid epidermotropic growth; cells of variable size; angiocentricity and angioinvasion common	CD3+ CD8+<br>CD4-<br>beta F1+<br>Granz.B+<br>perforin+<br>TIA1+<br>CD7+/-<br>CD5- CD2-<br>CD30- often<br>EBER-	TCR+	Poor
**PCGD-TCL**	Generalized skin lesions (patches, plaques, tumors, often in the extrimities)	Extranodal sites often; rare in lymph nodes, spleen and BM	Three major patterns:<br>-epidermotropic<br>-dermal<br>-subcutaneous<br>(often more than one pattern is present); cells of variable size	TCR-gamma+<br>Beta F1-<br>CD3+ CD2+<br>CD4- CD8-<br>(rarely CD8+)<br>CD5- CD7+/-<br>CD56+<br>Granz.+ TIA1+<br>perforin+<br>EBER-	TCR gamma+<br>TCR delta+<br>TCR beta-	Poor

**Primary cutaneous acral CD8+ T-cell lymphoma**	Isolated reddish papule or nodule; ear (most frequent); nose, foot	No	Dense, monotonous dermal and hypodermal infiltrate of medium sized cells; epidermis spared	CD3+ CD8+ TIA1+ beta F1+ TIA1+ (dot staining) CD4-; CD2+ CD5+ CD7+ (more often); Granz.B- and perforin- (often); CD56- CD57- TdT- CD30- CD10- BCL6- PD1- CXCL13- Ki67 low EBER-	TCR gamma+	Good; remission of surgery or radiotherapy; no need of CT
**Primary cutaneous CD4+ small/medium T-cell LPD**	Slow- growing solitary plaque or nodule; face, neck or upper trunk often No patches	No	Dense nodular dermal and hypodermal infiltrate with absent or focal epidermotropism; prevalence of small/medium cells; large cells <30%	CD3+ CD4+ PD1+ BCL6+/- CXCL13+ CD10- CD8- CD30- CD7- loss of T-cell antigen rare; TIA1- Granz-B- perforin- KI67 low	TCR+	Excellent; intralesional steroid, surgery or radiotherapy

Legends: BM: bone marrow; C-ALCL: cutaneous anaplastic large cell lymphoma; CT: chemotherapy; DLBCL: diffuse large B-cell lymphoma; EBVMCU: EBV-positive mucocutaneous ulcer; epid: epidermotropic; Granz: granzyme B; IHC: immunohistochemistry; IS: immunosuppression; LPD: lymphoproliferative disorder; LyP: lymphomatoid papulosis; MF: mycosis fungoides; PCGD-TCL: primary cutaneous gamma delta T-cell lymphoma; RSC: Reed-Sternberg cell
